# A technique for reducing patient setup uncertainties by aligning and verifying daily positioning of a moving tumor using implanted fiducials

**DOI:** 10.1120/jacmp.v9i4.2766

**Published:** 2008-10-30

**Authors:** Christopher Nelson, Peter Balter, Rodolfo C. Morice, Bum Choi, Rajat J. Kudchadker, Kara Bucci, Joe Y. Chang, Lei Dong, Susan Tucker, Sastry Vedam, Tina Briere, George Starkschall

**Affiliations:** ^1^ Departments of Radiation Physics The University of Texas M.D. Anderson Cancer Center Houston Texas U.S.A.; ^2^ Pulmonary Medicine The University of Texas M.D. Anderson Cancer Center Houston Texas U.S.A.; ^3^ Radiation Oncology The University of Texas M.D. Anderson Cancer Center Houston Texas U.S.A.; ^4^ Bioinformatics and Computational Biology The University of Texas M.D. Anderson Cancer Center Houston Texas U.S.A.

**Keywords:** implanted fiducials, image‐guided patient setup, lung tumors, kilovoltage imaging, cine imaging

## Abstract

This study aimed to validate and implement a methodology in which fiducials implanted in the periphery of lung tumors can be used to reduce uncertainties in tumor location.

Alignment software that matches marker positions on two‐dimensional (2D) kilovoltage portal images to positions on three‐dimensional (3D) computed tomography data sets was validated using static and moving phantoms. This software also was used to reduce uncertainties in tumor location in a patient with fiducials implanted in the periphery of a lung tumor.

Alignment of fiducial locations in orthogonal projection images with corresponding fiducial locations in 3D data sets can position both static and moving phantoms with an accuracy of 1 mm. In a patient, alignment based on fiducial locations reduced systematic errors in the left–right direction by 3 mm and random errors by 2 mm, and random errors in the superior–inferior direction by 3 mm as measured by anterior–posterior cine images.

Software that matches fiducial markers on 2D and 3D images is effective for aligning both static and moving fiducials before treatment and can be implemented to reduce patient setup uncertainties.

PACS number: 81.40.Wx

## I. INTRODUCTION

Provided that the spatial relationship between the lasers in the treatment room and the radiation isocenter of the treatment machine is accurately maintained, alignment of patient surface marks with the lasers should accurately align the patient treatment isocenter with the radiation isocenter of the treatment machine. The patient is not a uniform rigid body and respiratory motion, cardiac motion, and movement of the skin marks introduce uncertainties into the position of the patient's isocenter. To verify the accuracy of the isocenter position, the patient undergoes procedures such as kilovoltage (kV) or megavoltage (MV) imaging.^(^ ^1^ ^–^ ^3^ ^)^

Although surface marks are used for patient setup, the anatomy being treated is typically located deep within the patient. When setting up the patient using external markers, sufficient margins are added to the clinical target volume (CTV) to account for daily variations in the relative position of the tumor with respect to the surface marks.^(4)^ Ideally, the margin that accounts for these variations in tumor location is minimized to reduce excess toxic effects to normal tissue. However, small margins increase the possibility of a geometric miss of the CTV.

On the other hand, if three‐dimensional (3D) information on internal patient anatomy can be acquired before treatment, adjustments can be made in patient position based on internal anatomy rather than on surface marks.^(^ ^5^ ^–^ ^10^ ^)^ Basing patient setup on internal anatomy rather than on external markers should reduce the uncertainties in patient setup.

Patient setup based on internal anatomy requires acquisition of images of various anatomic regions at the time of treatment, which are then registered to the corresponding digitally reconstructed radiographs (DRRs) generated from the planning computed tomography (CT) scan. Several methods of image registration are used, including 3D‐3D image registration, two‐dimensional (2D)‐3D image registration, and 2D‐2D image registration.^(11)^ For 3D‐3D image registration, one 3D data set (typically CT, positron‐emission tomography, or magnetic resonance) is registered to a second 3D data set. This registration uses information in all three dimensions. A 2D‐2D alignment typically registers a pair of 2D images (either kV or MV portal images) to a pair of DRRs generated using ray tracing through the 3D data set. A 2D‐3D alignment registers fiducial marker locations in a pair of 2D images to the location of the fiducial makers in a 3D data set.

A 3D‐3D registration provides the most information for the registration, and it may be necessary for patient alignment in hypofractionation—that is, conditions in which tolerances for misalignment must be kept negligible. However, when radiation is delivered over many fractions, acquisition of 3D data sets each day during treatment is impractical. Thus 2D‐3D and 2D‐2D image registration techniques are most often used for conventional fractionation schemes. The disadvantage of using these techniques is that all the information in one ray passing through the 3D data set is stacked into one pixel in the 2D projection image. The assumption is that all structures in each image are located in two planes that intersect at the isocenter. Thus, 3D information on the patient is compressed into two planes, resulting in loss of information. Compounding the problem is the need to reproducibly identify the tumor (or surrogates for the tumor) in each image.

If fiducials are implanted near or in the tumor, they can easily be located and used for alignment. Using software to determine the location of fiducials in 2D images, the 3D coordinates of implanted fiducials from a pair of projection images can be compared with the 3D coordinates of the fiducials at the time of simulation, and shifts in couch position can be determined to best align the fiducials with their positions at simulation.

Compounding the problem from the standpoint of both imaging and accuracy in patient alignment is the movement of tumors in the lung and thoracic region with respiration.^(12)^ ^,^ ^(13)^ Two methods for reducing the effects of respiratory motion are delivery of radiation while the patient executes a breath‐hold maneuver and delivery of radiation synchronized to the patient's respiratory cycle (respiratory gating).^(14)^ ^,^ ^(15)^ These two methods minimize the effects of motion during treatment delivery, but alignment of a moving target is a much more complicated task than is alignment of a nonmoving target.

An ideal approach is based on using four‐dimensional (4D) imaging at simulation to acquire an accurate snapshot of tumor motion.^(16)^ The moving tumor can be delineated to generate an internal target volume (ITV). Each day at the treatment machine, some form of respiration‐correlated image‐guided patient setup could be used to ensure

- that the tumor is in the same position it was at simulation, and
- that the tumor motion is similar in both magnitude and direction to the motion observed at the time of simulation.

To accomplish these ends, 4D volumetric imaging capabilities or other volumetric imaging capabilities in combination with fluoroscopy could be used. Such capabilities are not yet clinically available at our institution. Thus, one practical approach to alignment of a moving target is to implant fiducials near the tumor and to use 4D imaging for target delineation. The fiducials can be easily and accurately located in a single phase of the 4D CT data set acquired at simulation, and alignment of the moving tumor and anatomy is based on fiducial locations in respiratory‐gated kV images. Finally, to coincide with the gated imaging for patient setup, treatment is delivered using respiratory gating. The gating system that enables both imaging and beam delivery uses externally placed markers (on the patient's abdomen) to monitor respiration (RPM: Varian Medical Systems, Palo Alto, CA).

The present work aimed to develop, validate, and implement a procedure that can be used to align moving tumors in the lung. To this end, we first determined the accuracy of a software package (Isoloc: NMPE, Inc., Lynnwood, WA) that can be used for online correction of patient setup based on the locations of implanted fiducials in daily kV projection images. The procedures thus developed were implemented in a patient to demonstrate their practicality.

## II. METHODS AND MATERIALS

### A. Phantom study

To validate patient setup using image guidance, we used a thoracic anthropomorphic phantom (Rando phantom: The Phantom Laboratory, Salem, NY). Cylindrical gold fiducials (0.9×3 mm: NMPE, Inc.) were inserted in the phantom.^(17)^ Insertion involved drilling small holes in thermoluminescent dosimeter inserts and securely placing the fiducials in these inserts. The inserts were then placed in the phantom.

Once the fiducials were placed in the phantom, we performed a CT simulation. Respiratory motion was simulated using a programmable moving platform.^(18)^ A stepping motor drives the platform, which moves in two dimensions by rolling up and down a ramp, thus translating the linear phantom motion into two dimensions. The phantom was aligned so that its sagittal axis was placed in the direction of motion of the CT table, in the same manner as a patient would be oriented. Three markers were placed on the surface of the phantom at the point where positioning lasers intersected the surface of the phantom, ensuring that all the markers were in the same superior–inferior (SI) plane. Before 4D CT image acquisition, the movable platform was started, and the phantom began translating. The particular motion that was simulated was a normal sinusoidal breathing motion at a rate of 12 breaths per minute, a frequency typical of patient respiration. When describing the motion of the platform, breaths per minute is used to describe the period with which the stepping motor oscillates. To acquire the 4D CT images, a reflective marker box was placed on the surface of the phantom, and the position of the marker box was monitored by RPM. Once the respiratory monitoring system was able to track the position of the box, the 4D CT (Discovery ST: General Electric Healthcare Systems, Milwaukee, WI) image set was acquired using a technique that retrospectively sorted images into 10 phase bins.^(19)^ Following image acquisition, the CT data sets were exported into a commercial radiation treatment planning system (Pinnacle^3^: Philips Medical Systems, Milpitas, CA). In the treatment planning system, an isocenter was arbitrarily chosen, and the coordinates of this isocenter were recorded. Then the coordinates of each implanted fiducial were recorded at the 50% phase (representative of end expiration).

A commercially available 2D−3D marker‐matching software program (Isoloc) was used for aligning both the phantom and the patient before treatment delivery. The coordinates of the isocenter and the three fiducial locations were imported from the treatment planning system into the 2D−3D marker‐matching program. The program loads anterior–posterior (AP) and lateral kV images taken from the imager on the treatment machine (Trilogy: Varian Medical Systems) and displays the images so that the fiducials can be located manually. Translational displacement can be computed using only one fiducial, but three fiducials are needed in the computation to account for rotations. In the present study only translational offsets from the simulation position were examined. Once the two kV images were acquired, in‐house software was used to embed a digital graticule into the image so that the isocenter could be identified. (No physical graticule is available for kV imaging on our system.) Once the two kV images were acquired, the fiducials and the isocenter were located interactively. The program then computed the translation needed to minimize the differences between the current fiducial locations and their respective locations at simulation.

To validate the 2D−3D marker‐matching software, we performed a series of tests. A preliminary test verified the accuracy of the couch readout. After the couch readout was verified, the next test validated that a point designated as the isocenter by the treatment planning system coincided with the true location of that point in the phantom. For this validation, the coordinates of a fiducial were entered into the 2D−3D marker‐matching software, identifying this point as the isocenter. We were thus able to use the crosshairs of the imager to visualize the fiducial at isocenter. The phantom was placed on the treatment couch, and AP and lateral kV images were acquired. Using the 2D−3D marker‐matching software, fiducials were located in the images and shifts in couch position required to move the chosen fiducial to isocenter were identified. The phantom was shifted as indicated by the software, and a second pair of images was acquired to verify the alignment. Once a fiducial was chosen as isocenter, it was clearly seen that the software accurately computed the shifts needed to align the phantom isocenter to the treatment machine isocenter because the fiducial was located directly under the graticule in both the AP and lateral images.

After we verified visually that the 2D−3D marker‐matching program could accurately shift the phantom to isocenter, another test validated the ability of the 2D−3D marker‐matching software to accurately quantify translations from isocenter. The phantom was aligned to an arbitrary isocenter, then the couch was shifted in increments of 1 cm. Images were acquired, and the 2D−3D marker‐matching software was used to calculate the shifts in couch position. The calculated shifts were compared to the actual couch shifts. Following acquisition of these images, the phantom was placed on the motion‐simulation platform and the same procedures were repeated; this time, image acquisition was triggered by the respiratory monitoring system.

### B. Image‐guided setup on a patient

After the marker‐matching software was validated by the phantom tests, our procedures were implemented on a patient. The patient, an 88‐year‐old man with stage III non‐small‐cell lung cancer, was accrued on an institutional review board–approved protocol (ID03‐0208: G.S., principal investigator).

The patient was treated definitively for lung cancer, receiving radiation treatment for 35 fractions in accordance with the protocol. Fiducials were implanted bronchoscopically in the periphery of the tumor.^(17)^ For this particular patient, 5 fiducials were implanted, but 2 fiducials had dislodged by time the simulation was performed. The patient underwent simulation with arms overhead; a vacuum bag (Vacloc: MedTec, Orange City, IA) and wing‐board and T‐bar combination (MedTec) were used for upper body immobilization. A 4D CT data set acquired at simulation was used to delineate the ITV (motion accounted by verifying contours on all 10 phases), and the fiducials were located in the 50% phase of the 4D CT data set. Note that, although the patient was receiving respiratory‐gated treatment, the ITV encompassed the entire extent of motion at the time of simulation. No reductions in margins were implemented because of the gated treatment or the image‐guided patient setup. The patient was treated with 5 fields, 4 of which were intensity‐modulated; an AP field was not intensity‐modulated so that the fiducial locations, at least in the SI and lateral directions, could be monitored during the gated treatment.

Each day before treatment, orthogonal kV images for alignment were acquired under respiratory gating at expiration. The gating window was chosen using a phase‐based amplitude‐triggered approach. After observing normal respiration, the gating window was visually adjusted so that it was positioned at end expiration (lowest position in the trough). The width of the window was adjusted so that no more than 2 – 3 mm of motion (according to the respiratory monitoring system) would occur during the beam enable period. During kV image acquisition, the image would be acquired as soon as the respiratory trace entered into the gating window. The alignment software was used to calculate the differences in the 3D position of the fiducials deduced from the kV images from their positions in the 50% phase of the 4D CT data set acquired at simulation. If the shifts needed to align the patient were greater than 3 mm in any one direction, the patient was translated to correct for the misalignment (rotations were ignored). If patient offset was more than 3 mm in one direction, and less than 3 mm in the other two directions, corrections were applied in all three directions. Following each shift in couch position, the patient was re‐imaged to verify the positioning. The alignment process was repeated until offsets in all three directions were less than 3 mm.

During treatment, MV images of the AP field were acquired in cine mode; 4 to 10 images were acquired every second to monitor fiducial location during delivery of the gated treatment. The gating window chosen for treatment was at the same level and width as that used to acquire the gated kV setup images. Once the patient's respiratory signal entered into the gating window, images were acquired continuously until the respiratory signal left the gating window. Several images were acquired per respiratory gate. Images were exported for analysis where each of the visible fiducials was located. These fiducial locations with respect to the treatment edges were identified and compared relative to their respective locations in the DRR generated from the 50% phase of the 4D CT acquired at simulation. Because cine images were acquired only for the AP field, the translations needed each day to align the patient before treatment could be applied to the fiducial locations in the MV cine images (in the direction opposite to the translation) to simulate where the fiducials would have been, had image guidance not been used to align the patient before treatment. For example, if the couch was translated by 7 mm to the right, 5 mm toward the gantry, and 3 mm down before treatment, the fiducial locations in the MV cine AP images would be translated *back*, 7 mm to the left and 5 mm out, which effectively positions the fiducials in their respective locations before image guidance. If multiple shifts were required to position the patient (that is, if the patient was imaged and repositioned, and if, upon re‐imaging, the shifts needed to align the patient were still larger than 3 mm in any direction), the net translation (deduced from multiple shifts) from the initial alignment was applied to simulate patient setup without image guidance.

To determine if setup uncertainties were reduced by the alignment technique, we quantified both the systematic and random errors of the patient setup on the basis of the locations of the fiducials in the MV cine images. To minimize the effects of acquiring varying numbers of images each day, the average fiducial location in the left–right (LR) and SI directions was determined for each treatment day, and this position was the one used to quantify random and systematic error alike. The systematic error was the difference between the mean position of all fiducial locations and the intended position (that is, at the time of simulation). The random component of setup error was quantified by taking the standard deviation of all fiducial locations.^(20)^ A *t*‐test was used to indicate statistical significance in the difference between the systematic error in image‐guided patient setup and that in simulated patient setup based on skin marks. Because standard deviation is the metric of evaluation of the random component of setup errors, an *f*‐test was used to indicate statistical significance. Values of *p* less than 0.05 indicated a statistically significant difference between image‐guided alignment and simulated skin mark alignment.

## III. RESULTS

### A. Phantom study

**Figure 1:**
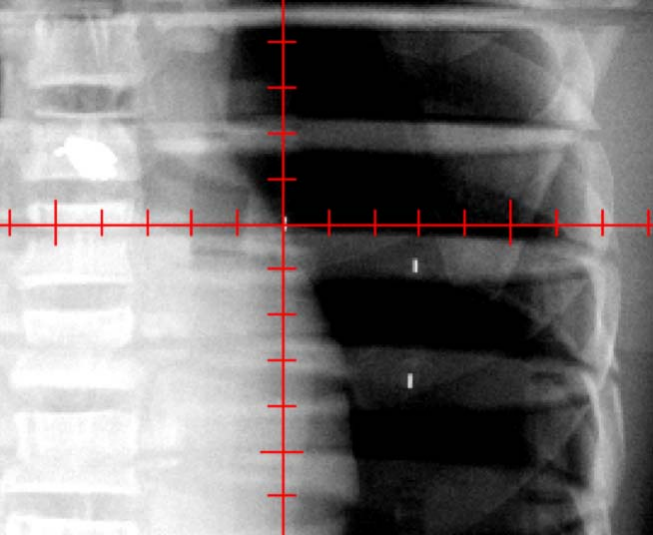
Anterior–posterior image displaying a fiducial, which was chosen as isocenter, at the center of the crosshairs that corresponds to isocenter.

The first validation was undertaken to determine whether the 2D−3D marker‐matching software could accurately shift the phantom to the isocenter. For this test, the chosen isocenter was a fiducial location. Fig. 1 is the AP image acquired with the isocenter chosen to be at the fiducial location. In this image, the fiducial is located directly over the crosshairs (representing the isocenter). It is easily seen that the 2D−3D marker‐matching program accurately shifted the phantom into the isocenter position (visualized using the fiducial location). Table 1 compares the couch translations with the translations determined by the software.

**Table 1:** Couch translations (relative to isocenter) and shifts determined by two‐dimensional–to–three‐dimensional marker‐matching software for images acquired using a static phantom

*Couch translation (cm)*			*Translation determined by software (cm)*		
*Vert*	*Long*	*Lat*	*Vert*	*Long*	*Lat*
−1.0	−1.0	1.0	−1.0	−1.0	1.1
1.0	1.0	−1.0	1.0	0.9	−0.9
−2.0	−2.0	2.0	−2.0	−2.1	2.1
2.0	2.0	−2.0	2.1	1.9	−2.0

Vert=vertical direction (up–down); Long=longitudinal direction (in–out); Lat=latitude direction (left–right).

After the static images were acquired, gated images were acquired using the motion platform. A pair of gated images was acquired, and the phantom was positioned at isocenter. As in the previous acquisition, the couch was translated in 1‐cm increments and images were acquired. Shown in Table 2 are the shifts in couch position and the computed shifts in couch position for the images acquired with the moving phantom.

**Table 2:** Couch translations (relative to isocenter) and shifts determined by two‐dimensional–to–three dimensional marker‐matching software for gated images acquired using a moving phantom

*Couch translation (cm)*			*Translation determined by software (cm)*		
*Vert*	*Long*	*Lat*	*Vert*	*Long*	*Lat*
−1.0	−1.0	−1.0	−0.9	−1.1	−1.0
1.0	1.0	1.0	1.0	1.0	1.0
−2.0	−2.0	−2.0	−2.0	−2.1	−2.0
2.0	2.0	2.0	2.1	2.0	2.1

Vert=vertical direction (up–down); Long=longitudinal direction (in–out); Lat=latitude direction (left–right).

Examination of Tables 1 and 2 reveals that the 2D−3D marker‐matching program computed the shifts in couch position needed to align the phantom at isocenter with an accuracy within 1 mm in each direction for images acquired using a static and a moving phantom. The ability of the 2D−3D marker‐matching software to accurately determine the shifts needed to move the phantom to isocenter is limited by the accuracy of locating the fiducials in the CT data set (2.5‐mm slice thickness). These are best‐case scenarios, given that the fiducial orientation is ideal, the distance between the fiducials is fixed, and the patient's anatomy is both small and not complicated. In a real patient, none of these conditions may be true.

### B. Image‐guided setup on a patient

**Figure 2:**
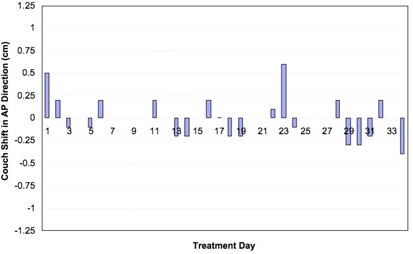
Shifts needed to align the patient in the anterior–posterior (AP) direction for each day of treatment.

**Figure 3:**
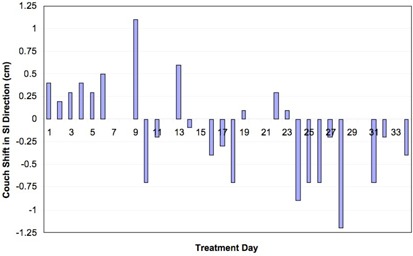
Shifts needed to align the patient in the superior–inferior (SI) direction for each day of treatment.

**Figure 4:**
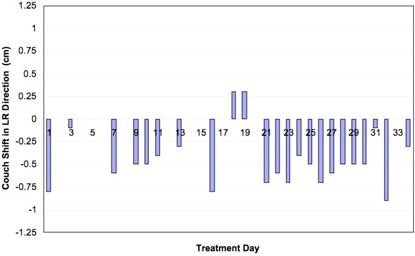
Shifts needed to align the patient in the left–right (LR) direction for each day of treatment.

Figs. 2, 3, and 4 show net shifts in couch position in the AP, SI, and LR directions needed to align the patient before treatment delivery when the patient was aligned using skin marks. For this particular patient, 2 of the 35 fractions were delivered on a different treatment machine that does not have the same imaging capabilities as the original machine. For those 2 days, the patient was aligned using the vertebral bodies in the MV portal images. No cine data were collected for those particular treatment days, and thus those days are excluded from the analysis. Recall that, if multiple shifts were needed to align the patient, only the net shift from the initial setup is reported. Multiple shifts were needed to align the patient on 7 of 33 treatment days. The patient was misaligned by more than 3 mm in at least one direction for 26 of 33 fractions—that is, the patient was adequately aligned (within 3 mm in all three directions on the first pair of images) for only 7 fractions; on the 2 days during which the patient was imaged and aligned by vertebral bodies, the physician indicated shifts of more than 0.5 cm each day, although those shifts are not included in the analysis.

Shifts of 7 mm or more in any one direction were detected in 13 of the 33 fractions. The maximum translations needed to align the patient in each direction were 9 mm, 12 mm, and 6 mm for the LR, SI, and AP directions respectively. The average (±1σ) couch translations over the course of treatment were −0.31 ±0.33cm, −0.09 ±0.47cm, and 0.0 ±0.2cm for the LR, SI, and AP directions respectively. We suspect that the large shifts were a result of imaging a moving fiducial in slightly different phases on a daily basis, in combination with the 3‐mm systematic error observed over the course of treatment in the LR direction.

**Figure 5:**
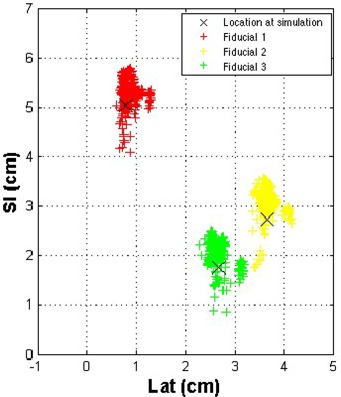
Fiducial locations as determined in the anterior–posterior field in gated megavoltage cine images. SI=superior—inferior direction; Lat=lateral direction.

Cine images were acquired of the patient's AP field during delivery of gated treatment. The fiducial locations were determined with respect to the treatment field edges and are illustrated in Fig. 5. Overlaid on this figure is the location of each of the fiducials on the DRR generated from the 50% phase of the 4D CT data set acquired at simulation. Because the patient was aligned on the basis of fiducial locations in the gated kV images, the fiducial locations in the MV images can be translated by the same amount that was used to translate the patient before treatment so as to examine where the fiducials would be located if the patient had not been aligned before treatment.

**Figure 6:**
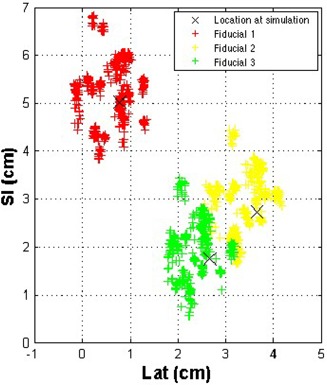
Theoretical fiducial locations as determined in the anterior–posterior field in gated megavoltage cine images were derived from the same data shown in except that the fiducial locations for that particular day are translated in the opposite direction, at the same magnitude in the left–right and superior–inferior directions as that used to align the patient before treatment. SI=superior—inferior direction; Lat=lateral direction.

**Figure 7:**
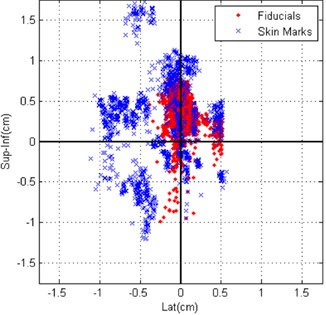
Locations of all three fiducials displayed so that the origin was the simulation location for alignment based on skin marks and based on fiducial locations. Sup‐Inf=superior‐inferior direction; Lat=lateral direction.

Fig. 6 shows the theoretical fiducial locations if the patient were positioned only on the basis of external skin marks, rather than on implanted fiducials. A qualitative comparison of Figs. 5 and 6 shows that patient setup using image guidance based on fiducial locations reduces setup uncertainties. Shown in Fig. 7 are the locations of all three fiducials, normalized so that the fiducial locations are centered on their location at simulation. Because multiple images were acquired each day, quantification of setup uncertainties and minimization of the effects of different days having different numbers of fiducial locations necessitated determination of the average fiducial location in the LR and SI directions each day for each fiducial. (Recall that a reduction in setup uncertainties is determined from the cine images acquired during the AP field.)

As measured on the MV cine images, the systematic setup errors (difference in mean fiducial position from simulation) averaged over all three fiducials using the simulated skin mark alignment in the LR and SI directions were 3 mm and 2 mm respectively. Based on the real data when the patient was aligned using fiducials, the systematic component of setup error was reduced to less than 0.5 mm in the LR direction (p<0.01 by *t*‐test) and 3 mm in the SI direction. This error was greater than the error caused by simulated skin mark alignment; however, because we used 3 mm as our action level, we expect that this error is within the uncertainties of aligning a moving target. Furthermore, at least 1‐mm uncertainty is present in our measurement, and so we can conclude that the systematic component of setup uncertainties was not significantly reduced. The random component of setup error, which is essentially the daily variation in positioning (quantified by the standard deviation), was reduced from 4 mm to 1 mm in the LR direction (p<0.01 by *f*‐test) and from 6 mm to 2 mm in the SI direction (p<0.01 by *f*‐test) when the patient was aligned using fiducials rather than skin marks.

## IV. DISCUSSION

Our results show that we were able to reliably align both static and moving targets by using kV imaging to localize fiducials in orthogonal images (within 1 mm). This image‐guided technique was successfully implemented in a patient, with significant reduction in patient setup uncertainties, especially in the random component, which comprises daily variations in position.

Fiducials implanted in the prostate have been used extensively for patient alignment before treatment.^(21)^ ^,^ ^(22)^ For lung tumors, Shirato et al.^(7)^ ^,^ ^(8)^ have extensively used fiducials implanted near the tumor for both patient alignment and gated delivery of the treatment beam. They used fiducial locations to align patients before treatment and to deliver gated hypofractionated treatment based on the locations of the fiducials in fluoroscopic images. In one such study, these investigators quantified the amplitude of fiducial motion and the technique of 4D patient setup.^(23)^ They did not, however, report setup uncertainties based on fiducial locations.

Setup uncertainties as measured by the locations of implanted fiducials for conventionally fractionated treatments were recently reported by Nelson et al.^(17)^ In that study, setup uncertainties were measured at 0.4 cm and 0.6 cm for the LR and SI directions respectively for random errors and for systematic errors; positional uncertainties of 0.3 cm and 0.2 cm were measured during treatment for the LR and SI directions respectively. Setup uncertainties quantified by patient skin marks in a simulated setup were similar to those that we previously measured, which was expected, because the same immobilization was used in the present study as in the previous study. As in the Nelson et al.^(17)^ study, we observed fiducials dislodging from the patient shortly after implantation. This dislodgement is probably a result of the bronchoscopic implantation technique, which has been observed to have a lower incidence of pneumothorax relative to percutaneous fiducial implantation.^(24)^ ^,^ ^(25)^ The two fiducials that remained and that were used for alignment were located several centimeters apart on the edge of the tumor and were monitored by weekly 4D CT.

In a feasibility study by Willoughby et al.,^(26)^ fiducials were percutaneously implanted near lung tumors in 11 patients. These investigators used fluoroscopic tracking of the fiducials in synchronization with external markers to deliver a gated treatment. However, because of their technique of percutaneous fiducial implantation, the incidence of pneumothorax was relatively high. Other studies have reported setup uncertainties for treatment of lung tumors without fiducials.

In a study by de Boer et al.,^(27)^ which used an offline correction protocol to reduce systematic positioning errors, systematic errors in 40 patients were reduced to less than 2 mm in each direction, and random errors were reduced to approximately 1 mm. Without the offline correction protocol, these errors were larger.

In our study, image‐guided setup reduced the systematic component of the setup error substantially in the LR direction, and the random component in both the LR and AP directions. No reduction in systematic error was observed in the SI direction, a result that can be attributed to the fact that we were imaging fiducials that moved more than 2 cm during normal respiration in addition to the uncertainties of the gating system. However, both components of patient setup error in our study are larger than those reported by de Boer et al. In their study, MV portal images and a template‐matching algorithm were used to compare the location of structures that do not move with respiration (vertebral bodies) with their location at the time of simulation; a minimum of five images was acquired per patient. We had only 1 patient, but we monitored fiducial locations for 33 fractions during the course of treatment. Even after acquisition of gated kV images and use of the locations of the fiducials in these images each day for patient alignment, setup uncertainties were not negligible.

Our study is not without its limitations. One major limitation is our imaging capabilities. In the present study, we acquired a gated kV image, rotated the gantry by 90 degrees, and acquired a second gated kV image. An ideal system would be one in which simultaneous, gated orthogonal kV images could be acquired.^(10)^ ^,^ ^(28)^ Uncertainties in the gating system further compound the problem. For many fractions, the fiducial locations in the AP and lateral kV images were clearly not in phase. This problem would have been eliminated if dual simultaneous kV imaging capabilities were available. Furthermore, the correlation during gated treatment of tumor position with external skin surface position is still not well understood. Large intrafractional changes in patient respiration also limit our system, in that the patient is aligned only prior to treatment and is not verified until completion of treatment (analysis completed retrospectively).

Although the imaging capabilities are readily available and implantation of the fiducials by bronchoscopy is a relatively simple outpatient procedure (insertion takes approximately 20 minutes in our institution), the invasiveness of the procedure itself is a drawback. Pneumothorax and other short‐term side effects have not been observed for the patient in this study and for others in our institution that have thus far undergone bronchoscopic fiducial implantation. We believe, however, that if the fiducials are near the tumor, the resulting significant reduction in patient setup uncertainties outweighs the negative effects of the implantation procedure.

Regardless of the technique used to align lung tumors before treatment, all uncertainties must be considered and carefully incorporated into the planning target volume margins. Although setup uncertainties were reduced in a patient by using 3 mm as an action level, we observed fiducial locations to be spread over approximately 1 cm in each direction. For this particular patient, non‐gated intrafractional fiducial motion approached 2 cm as measured by serial 4D imaging.

Alignment to a moving target is a challenging problem, and the technique for doing so is still in development. There is room for improvement. Setup uncertainties and respiratory motion both displace the tumor from the intended location, and thus extreme caution should be used if setup margins are reduced without correcting for patient positioning.
